# The change of a district hospital antimicrobial consumption in and after national antimicrobial appropriate use intervention, China

**DOI:** 10.1186/2047-2994-4-S1-P179

**Published:** 2015-06-16

**Authors:** Y-X Chen, L-J Zhao, B-Y Liu, J-L Cao, D-M Dong, L-L Chen, S Feng, B Gao

**Affiliations:** 1 Nosocomial Infection Control Unit, Wuqing District Hospital, China; 2 Pharmacy, Wuqing District Hospital, China; 3 Clinical Microbiologic lab, Wuqing District Hospital, China; 4 Faculty of Graduate Research, Tianjin Medical University, Tianjin, China

## Introduction

Clinical use of antimicrobial was promoted by a national special clinical antimicrobial appropriate use program (2011~2013) and legislation (in 2012) by the Ministry of Health in China.

## Objectives

To investigate the time-phased effectiveness of the national intervention to antimicrobial clinical use in a district hospital, China.

## Methods

Multi-strategy according to national special program and legislation to reasonable use of antimicrobial, including antimicrobial prescribe target setting, surveillance on prophylactic use of antimicrobial rate to clean wound surgery and surgical set infection, and, as well as performance feedback etc, were introduced in a district hospital since 2011. WHO Anatomical Therapeutic Chemical classifications of antimicrobial was used in calculated defined daily doses (DDDs). Annual antimicrobial consumption data from 2011.1 to 2014.12 were obtained and converted to DDDs per 1,000 patient-days.

## Results

The strength of antimicrobial consumption by DDDs per 1,000 patient-days was decreased in the hospital by 32.20%, 1745.940, 1143.471, 1144.949, and 1183.821, respectively from 2011 to 2014. About 70% of antimicrobials use was stably prescribed in its outpatient settings during the investigation (73.32% in 2011, 70.74% in 2012, 68.39% in 2013, and 68.47% in 2014). The strength of antimicrobial consumption by DDDs per 1,000 patient-days was decreased by 70.32% in the inpatient-units performing surgical procedure with clean wound, 623.819, 337.267, 276.622 and 224.563, respectively from 2011, meanwhile decreasing prophylactic use to class one wound, 42.28%(416/984 in 2011), 21.95%(373/1699 in 2012), 14.77%(288/1950 in 2013) and 14.24%(232/1629 in 2014). During the same time, the strength of antimicrobial consumption by DDDs per 1,000 patient-days was increased by 22.30% in the rest inpatient-units, 383.947, 332.685, 418.225 and 467.484, respectively.

## Conclusion

National intervention of antimicrobial judicious use was effective in outpatient clinic and units operating clean procedure in the district hospital.

## Disclosure of interest

None declared.

